# A Practical, Short, [^18^F]F-DOPA PET/CT Acquisition Method for Distinguishing Recurrent Brain Metastases from Radionecrosis Following Radiotherapy

**DOI:** 10.3390/jcm14072168

**Published:** 2025-03-22

**Authors:** Pascal Bailly, Roger Bouzerar, Ines Barrat, Mathieu Boone, Alexandre Coutte, Marc-Etienne Meyer

**Affiliations:** 1 Nuclear Medicine Department, Amiens University Hospital, 80000 Amiens, France; bouzerar.roger@chu-amiens.fr (R.B.); ines.barrat@gmail.com (I.B.); meyer.marc-etienne@chu-amiens.fr (M.-E.M.); 2 Medical Oncology Department, Amiens University Hospital, 80000 Amiens, France; boone.mathieu@chu-amiens.fr; 3 Radiotherapy Department, Amiens University Hospital, 80000 Amiens, France; coutte.alexandre@chu-amiens.fr

**Keywords:** [^18^F]F-DOPA PET imaging, short acquisition, brain metastasis, progression, radionecrosis

## Abstract

**Background/Objectives:** Determining whether 3,4-dihydroxy-6-[^18^F]fluoro-L-phenylalanine positron emission tomography/computed tomography ([^18^F]F-DOPA PET/CT) data indicate brain metastasis progression (MP) or brain radionecrosis (RN) is challenging. The aim of this study was to present a method usable in the clinical setting without dedicated software that relies on less than five minutes of SiPM PET/CT data collected immediately after [^18^F]F-DOPA injection. **Methods:** We prospectively enrolled 15 patients with 19 lesions. Each acquisition was conducted in list mode (LM) for 25 min using a four-ring SiPM PET/CT system. We reconstructed three volumes from the LM file: acquisition duration of 120 s (V120), 270 s (V270), and 10 min for the standard clinical volume (Vclin). We measured each lesion’s maximum voxel activity (LS_max_) and the corresponding mean activity with its standard deviation (CL_mean_, CL_sd_). We then calculated the LS_max_/CL_mean_ ratio and the coefficient of variation (COV), defined as 100 × (CL_sd_/CL_mean_). **Results**: Lesions were classified as RN (n = 7) and MP (n = 12). For all volumes, LS_max_/CL_mean_ differed significantly. The COV parameter exhibited significant differences in all comparisons except for V120 vs. V270. The best diagnostic performances were observed for V120 and V270, with an accuracy of 94.7%. The AUC values were 97.6% in both cases. **Conclusions**: A simple, static [^18^F]F-DOPA PET/CT acquisition, starting 1.5 min after injection and lasting less than five minutes, permitted reaching excellent diagnostic performance (100% sensitivity, 91.7% specificity, and 97.6% AUC) in discriminating between RN and MP.

## 1. Introduction

The prime source of brain metastases (BM) is primary lung cancer, followed closely by breast cancer and melanoma [1]. The treatment options for BM are conditioned by a number of factors, including the site, number, and size of the lesions. Surgical excision can be considered in select cases [2]. The therapeutic arsenal also includes stereotactic radiosurgery [3], chemotherapy [4], and immunotherapy [5]. Stereotactic radiosurgery is now one of the most common treatments for BM [6].

The area affected by radiation necrosis (RN) potentially induced by radiotherapy for BM treatment should be regularly monitored for metastasis progression (MP) [7]. The most common method for monitoring BM and differentiating between MP and RN is magnetic resonance imaging (MRI), which usually includes T2-weighted FLAIR and T1-weighted sequences with gadolinium contrast injection. On MRI, radionecrosis and recurrence may exhibit several overlapping features, such as central necrosis or perilesional edema. In addition, depending on the anatomical location, distinguishing enhancing metastasis from background vascular enhancement can sometimes be challenging [8,9]. Additional advanced functional MRI sequences, such as spectroscopy, diffusion, and perfusion imaging, can provide complementary information [10,11,12]. In particular, the latter technique, which can be performed using dynamic susceptibility contrast or dynamic contrast-enhanced imaging, can produce maps of relative cerebral blood volume, considered an indicator of local vascularization. These techniques are very useful in helping to differentiate between the two diagnoses, but each of these methods has inherent limitations from both technical and practical standpoints [9,10]. In fact, interpretation can be hampered by surrounding vessels or artifacts arising from anatomical locations, such as infratentorial areas where major susceptibility artifacts may be encountered. Furthermore, diagnostic thresholds are often influenced by the perfusion sequence parameters, the quality of the contrast bolus injection, and the specificity of the sophisticated post-processing software algorithms required to generate the perfusion maps. Consequently, the widespread integration of these techniques may be limited by the absence of standardization in imaging acquisition and post-processing analysis [9,10].

Positron emission tomography (PET) imaging with 3,4-dihydroxy-6-[^18^F]fluoro-L-phenylalanine ([^18^F]F-DOPA) is a valuable resource for the diagnosis and management of brain tumors [13]. Many studies of brain tumors have shown that [^18^F]F-DOPA PET/computed tomography (CT) imaging is equivalent or even superior to CE-MRI [14,15]. Nevertheless, only a limited number of research teams has explored the application of the [^18^F]F-DOPA PET/CT technique for BM [16]. The recommended protocol for imaging brain tumors with [^18^F]F-DOPA PET/CT is set to a 15 min period for tracer uptake post-injection, followed by a 10 min acquisition (i.e., clinical volume) [17].

The latest silicon photomultiplier-based PET scanners (SiPM PET) are more sensitive than earlier models equipped with photomultiplier tubes [18]. Barrat et al. [19] demonstrated that early, dynamic [^18^F]F-DOPA PET/CT acquisitions on a SiPM PET machine could be used to differentiate between RN and MP, although dedicated software was required. The objective of the present study was to describe our simpler method (i.e., without the need for dedicated software), which is suitable for use in clinical practice and is still based on less than 5 min of acquisition of SiPM PET/CT data immediately after the injection of [^18^F]F-DOPA.

## 2. Materials and Methods

### 2.1. Study Population

The main inclusion criterion discussed at a multidisciplinary team meeting was diagnostic uncertainty with regard to MP or RN on MRI at least four months after the end of radiotherapy. This prospective study was approved by an independent ethics committee (Comité de Protection des Personnes Nord-Ouest IV, Lille, France; reference 2022-A00930-43, approval date on 16 May 2022).

All the study participants provided their informed consent and fasted for at least four hours before the [^18^F]F-DOPA SiPM PET/CT examination. None of the patients were premedicated with carbidopa.

The final diagnosis (MP or RN) was determined using either histopathology analysis of surgically excised tissue from the lesion or a clinical and radiological assessment by a multidisciplinary team up to six months after the [^18^F]F-DOPA PET/CT examination.

### 2.2. [^18^F]F-DOPA PET/CT Imaging

All acquisitions in the current study were conducted in the time-of-flight (TOF) mode using a 4-ring whole-body PET/CT system based on the SiPM technology (Discovery Meaningful Insights (DMI), General Electric (GE) Healthcare, Waukesha, WI, USA), hereafter referred to as DMI [20]. This PET system has axial and transaxial fields of view measuring 198 mm and 700 mm, respectively. DMI is equipped with 19,584 lutetium–yttrium–orthosilicate (LYSO) scintillator crystals (each measuring 5.3 mm × 3.95 mm × 25 mm) integrated into a 9 × 4 detector block. The LYSO crystals, combined with a Hamamatsu SiPM array, are paired with an application-specific integrated circuit (ASIC) developed internally by GE. As such, the output energy is digitized by an external analog-to-digital converter, and the synchronization signal is digitized by an external time-to-digital converter. The conversion of the photosensor signals provides energy, position, and time information for each detected event. The system’s energy window was set to 425–650 keV. The coincidence timing window was set to 4.9 ns, and the TOF information was acquired and encoded with a time resolution of less than 400 picoseconds full width at half-maximum (FWHM). DMI is equipped with a 64-slice CT scanner (Revolution™ EVO CT, GE Healthcare, Waukesha, WI, USA) capable of generating slices with thicknesses ranging from 0.63 to 10 mm, with rotation time increments from 0.35 to 1 s in steps of 0.1. The field of view has a diameter of 700 mm. Throughout the three-dimensional acquisition, the emission data (i.e., prompts = trues + randoms + scatter events) were recorded in real time in a list mode (LM) file composed of 8-byte words containing TOF information. For this study, data collected from a brain-centered step were stored in the LM file for 25 min, starting just before the [^18^F]F-DOPA injection.

Before reconstruction, the LM data were histogrammed (i.e., rebinned into sinogram files). For our DMI system’s one-step acquisition, the sinogram file corresponded to a four-dimensional array with 1261 × 29 × 415 × 272 bin detectors. All PET images were generated using the attenuation-weighted ordered subset expectation maximization algorithm (AW-OSEM) [21]. The reconstruction parameters were as follows: two iterations, 17 subsets, 256 × 256 matrix, a 30 cm useful field of view, and a 5 mm post-filter. Point spread, scatter, and attenuation corrections were also applied.

### 2.3. In-House MATLAB Software Tool

For the analysis of the LM file in this study, we developed software based on MATLAB (2023b, The MathWorks Inc., Natick, MA, USA). This allowed us to study the kinetics of fixation of the radiotracer (i.e., [^18^F]F-DOPA) in a voxel or in a set of voxels, as well as the temporal evolution of the counting statistics of the emission data (i.e., events) collected by the crystals.

We also developed an interface for this software that allows searching for the maximum value of a voxel in a spherical volume of interest and its coordinates, as well as for the manual placement by the user of a circular region of interest to calculate the average value in this area.

### 2.4. Experimental Volumes

**Figure 1:**
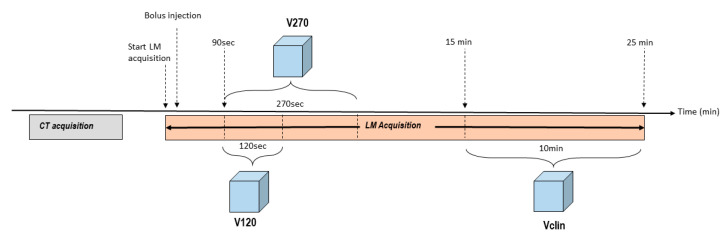
A schematic depiction of the timeline for CT acquisition, [^18^F]F-DOPA injection, and PET list mode acquisition, together with an overview of the generation of the V120, V270, and Vclin volumes used in the study.

We generated two volumes from the LM file, corresponding to the acquisition durations of 120 s and 270 s (Figure 1). These volumes are referred to hereafter as V120 and V270, respectively. In all the cases, the first 90 s of the LM file were discarded in order to compensate for (i) the lack of synchronization between the start of the acquisition and the manual [^18^F]F-DOPA injection, (ii) the influence of blood vessels close to the lesions during the first vascular passage, and (iii) the activity’s variable time of arrival in the camera’s field of view (which depends on patient-related factors, such as venous injection in the arm and the state of the circulatory system) [19].

### 2.5. The Clinical Volume

To reproduce the [^18^F]F-DOPA PET examination performed in routine clinical practice, we generated a clinical volume (referred to hereafter as Vclin) from the last 10 min of the LM file. Hence, the Vclin corresponded to 15 min of uptake after the injection and 10 min of acquisition as shown in Figure 1.

### 2.6. Measurement Analysis

The maximum voxel activity (${LS}_{max}$) of each lesion (LS) was measured. Furthermore, the mean activity and the SD were measured in a circular region of interest (diameter: 10 mm) manually positioned contralaterally (CL) to the same slices as the ${LS}_{max}$ (${CL}_{mean}$, ${CL}_{sd}$). The ${LS}_{max}/{CL}_{mean}$ ratio and the coefficient of variation were then calculated as follows:

(1) $$\mathrm{COV}=100\;\times \;\frac{{CL}_{sd}}{{CL}_{mean}}$$

### 2.7. Statistical Analysis

The categorical variables were reported as the frequency (percentage), and the quantitative variables were reported as the means ± SD or the median [interquartile range (IQR)]. For the mean values of the continuous variables, the MP and RN patient groups were compared using the Mann–Whitney U test. Reconstructions were compared with regard to the *COV* and SD values using Wilcoxon’s signed-rank test. The level of diagnostic performance was assessed using a receiver operating characteristic curve analysis, with maximization of Youden’s index. The sensitivity, specificity, accuracy, and area under the curve (AUC) were calculated.

Analyses were performed using Rstudio software (version 2023.09.1, running on R version 4.4.2 (https://www.r-project.org (accessed on 9 September 2023)). The threshold for statistical significance was set to *p* < 0.05.

## 3. Results

### 3.1. Patient Characteristics

Fifteen patients with a total of 19 lesions were included in the study. The patients’ characteristics are reported in Table 1. Seven of the 19 lesions were classified as RN, and the other 12 were classified as MP. Only three lesions had been surgically resected before radiotherapy. These three cases involved a 65-year-old woman with a primary breast tumor and a right cerebellar secondary lesion diagnosed as metastasis progression; a 75-year-old woman with a primary breast tumor and a left occipital secondary lesion diagnosed as radionecrosis; and a 70-year-old man with a primary lung tumor and a left frontal secondary lesion diagnosed as radionecrosis. The patients had undergone stereotactic radiotherapy (for n = 16 lesions), stereotactic radiosurgery (n = 2), or both stereotactic radiosurgery and whole-brain radiation therapy (n = 1). Metastases were irradiated with a median [IQR] dose of 30 [24–33] Gy. The median [IQR] activity concentration of [^18^F]F-DOPA injected was 1.9 [1.8–2.0] MBq/kg.

### 3.2. Dynamic Presentation of Concentrations and Counting Statistics

**Figure 2:**
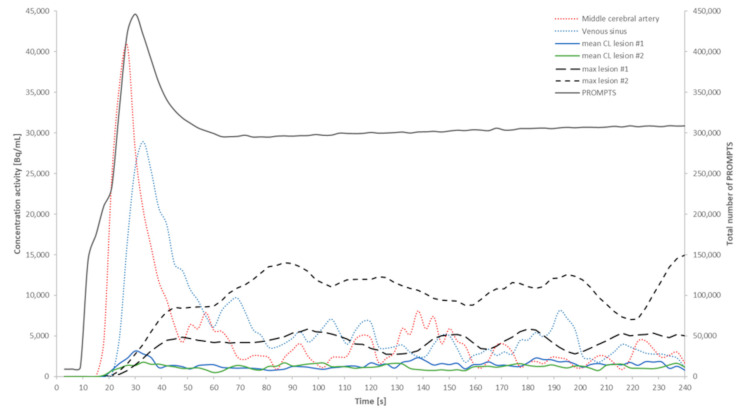
Temporal evolution of radiotracer concentrations obtained in different cerebral locations and number of emission data collected using DMI for the patient harboring two cerebral lesions. This graph comprises an X-axis, which represents the time interval in seconds, and two Y-axes, one displaying radioactive concentrations in the tissues (Bq/mL) and a secondary axis representing the number of prompts that DMI collected and stored in the LM file. For this patient being monitored for a radionecrosis (lesion #1) and a metastasis (lesion #2), we present the tracking of the concentration in a voxel located in the middle cerebral artery (red dotted curve) and in the venous sinus (blue dotted curve). We also present the maximum of lesion #1 (large dotted black curve) and the average activity in the corresponding contralateral region of interest (solid blue curve). Similarly, for lesion #2, the solid green curve corresponds to the average activity present in the contralateral region of interest, and the fine-dotted black curve represents the maximum value for this lesion. The solid black curve placed at the top of the graph corresponds to the number of events (i.e., trues + scatter events + randoms) collected by DMI during the acquisition phase.

In Figure 2, we present the dynamic results obtained for the patient acquisition with two lesions.

The arterial peak was reached at 27 s, while the venous peak occured at 33 s after the start of the acquisition. The initial horizontal plateau of the PROMPTS curve ended at 9 s after the start of the acquisition and reached a counting peak at 30 s, leveling off at a new plateau of 300,000 counts from the 66th second. The curves obtained for the two averages in the respective contralateral regions of interest, nearly overlapping, stabilized at approximately 1200 Bq/mL from 42 s onward. As for the two curves tracking the maximums of the two lesions, they appeared quite distinct, with the curve for lesion #2 consistently higher than that for lesion #1.

### 3.3. Quantitative Analysis of the Clinical and Experimental Volumes

**Figure 3:**
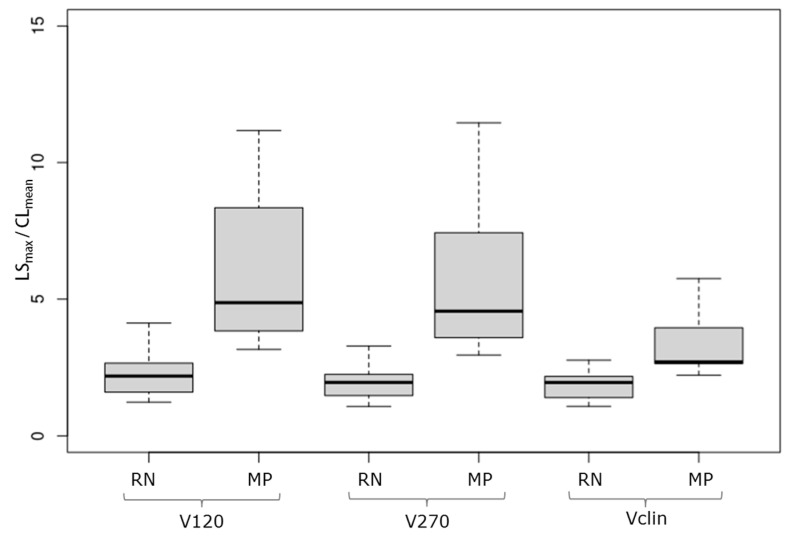
Boxplots of the LS/contralateral region of interest [^18^F]F-DOPA ratio for the V120, V270, and Vclin volumes in the RN and MP groups.

For all the reconstructed volumes, the RN and MP groups differed significantly with regard to ${LS}_{max}$/${CL}_{mean}$ (Figure 3 and Table 2).

The median values (95% CI) for *LS_max_*/*CL_mean_* were as follows:

(i) 2.19 (1.58; 2.80) and 4.87 (3.16; 11.17) for RN and MP, respectively, with regard to the V120 volume;
(ii) 1.96 (1.32; 2.30) and 4.56 (2.96; 11.46) for RN and MP, respectively, with regard to the V270 volume;
(iii) 1.95 (1.27; 2.28) and 2.70 (2.22; 5.75) for RN and MP, respectively, with regard to the Vclin volume.

**Figure 4:**
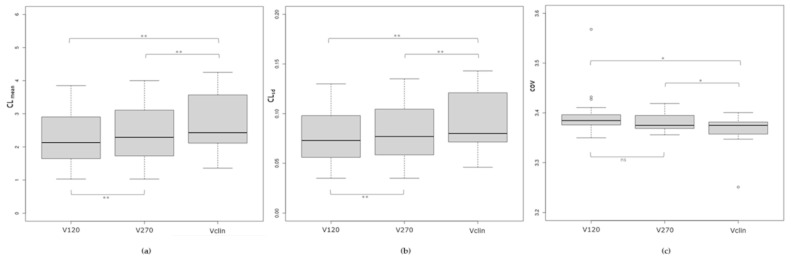
(**a**) Boxplots of the mean, (**b**) standard deviation, and (**c**) coefficient of variation of the [^18^F]F-DOPA values in the contralateral region of interest for the V120, V270, and Vclin volumes. Note: * *p* < 0.05; ** *p* < 10^−3^.

Comparisons of the reconstructed volumes revealed statistically significant differences in the ${CL}_{mean}$, ${CL}_{sd}$, and *COV* variables, except for the *COV* for V120 vs. V270 (Figure 4).

**Figure 5:**
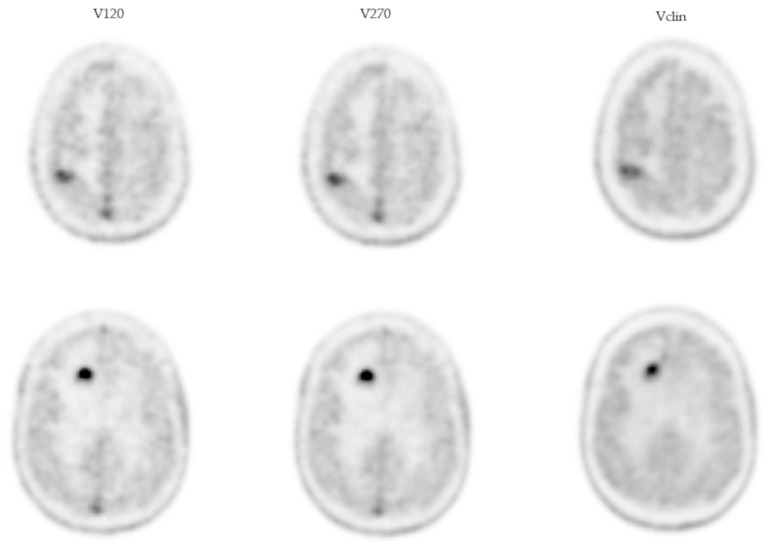
Illustrative [^18^F]F-DOPA PET/CT images of the patient presenting an RN (**top row**) and an MP (**bottom row**). The first, second, and third columns correspond to V120, V270, and Vclin, respectively.

**Figure 6:**
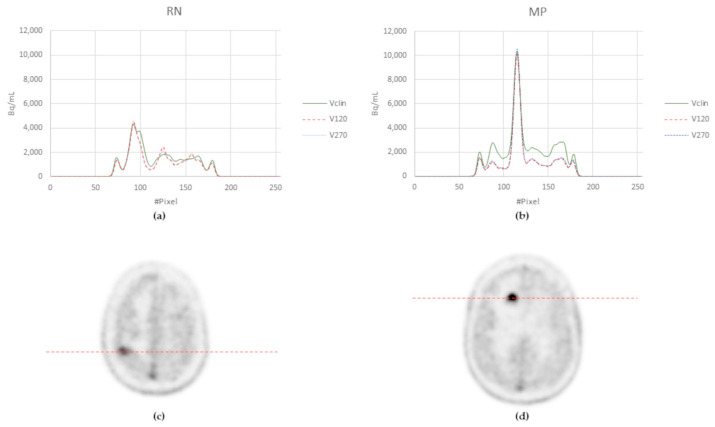
(**a**) Activity profiles for RN along the red dotted line (**c**) and for MP (**b**) along the dotted line (**d**) in V120, V270, and Vclin in the same patient.

Examples of the clinical, V120, and V270 images in the patient with both RN and MP are shown in Figure 5, and the corresponding activity profiles are shown in Figure 6.

**Figure 7:**
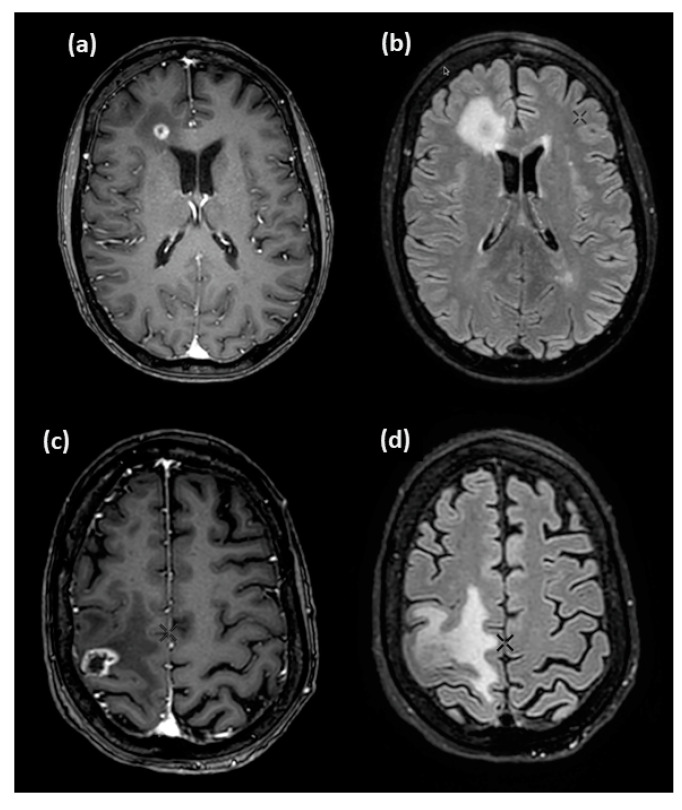
Illustrative MR images of the patient presenting an MP (**top row**) and an RN (**bottom row**). The slices from the 3D T1 contrast-enhanced (**a**,**c**) and 3D FLAIR (**b**,**d**) acquisitions show the MR appearance of the brain lesions corresponding to the PET images of the patient reported in Figure 5.

The MR images obtained from the 3D T1 contrast-enhanced and FLAIR sequences depicting the two lesions are shown in Figure 7.

### 3.4. Assessment of Diagnostic Performance

The best levels of diagnostic performance were observed for V120 and V270, with an accuracy of 94.7% for the ${LS}_{max}$/${CL}_{mean}$ ratio in both cases (Table 3). The AUC values were 97.6% in both cases.

The level of diagnostic performance was slightly lower for the Vclin volume, which was less specific (75%) and less accurate (84.2%).

## 4. Discussion

Depending on the number and location of the lesions, radiotherapy is the most common treatment for BM [22]. Post-radiotherapy follow-up is essential for establishing the differential diagnosis of RN vs. MP. Although Barrat et al. recently developed an effective, dynamic protocol for this differential diagnosis, its implementation required specific, in-house software and a nonstandard acquisition [19]. On the basis of this work, we sought to develop a simple, practical solution for routine clinical practice and, thus, a short, standard, static PET acquisition after the injection of [^18^F]F-DOPA. As a preliminary step, the use of in-house software was necessary to determine, regardless of the patient, the delay (i.e., 90 s) from the start of the LM file to ensure that the measurements (i.e., max) on the lesions (i.e., MP or RN) were not disrupted by the adjacent arteries or veins (Figure 2). Furthermore, we empirically studied two durations (i.e., V120 and V270), which, depending on the injected activity and thus influencing the amount of emission data collected, allowed for obtaining reliable measurements and, therefore, the sensitivity and specificity values comparable to the conventional clinical protocol.

Our method’s excellent ability to discriminate between RN and MP was similar to that reported by Barrat et al. [19]. In our study, the volume with the shortest acquisition duration (V120) was effective for this differential diagnosis. A short-duration PET/CT acquisition is more comfortable for patients and reduces the likelihood of head movement during the examination. A short but reliable acquisition relies on the use of highly sensitive SiPM-based PET/CT. A limitation in obtaining a PET image suitable for quantification is related to counting statistics [23], which are low with PET systems equipped with photomultiplier tubes [24]. To evaluate the impact of counting statistics on V120, V270, and Vclin when using identical reconstruction parameters, we measured the *COV* [25] and quantified the level of noise in the contralateral region of interest. Although the values were not equivalent in pairwise comparisons, they were still generally quite close to each other.

Clinicians commonly perform a semi-quantitative PET analysis by calculating the ratio between the lesion’s maximum value and the mean value measured in the corresponding contralateral region [26]. These measurements are known to be affected by the reconstruction algorithm used to generate the volumes [27]. To ensure applicability in all clinical centers, we used the AW-OSEM algorithm implemented in all commercially available PET cameras [21]. It should also be noted that the PET camera occupancy time for this method (with the injection under the camera) was nearly 4 min for V120 and 6 min for V270. For the Vclin reference protocol used in clinical practice, a 10 min acquisition is preceded by a 15 min wait period for radioelement uptake. Therefore, the method described here may be useful for increasing and optimizing the workflow.

Our study’s main limitation was the small sample size, which decreased the statistical power. This limitation was accentuated by the presence of outliers in the noise distribution. Lastly, the reference (histological) diagnosis was not optimal because only a small proportion of the patients underwent surgical resection due to the risks associated with neurosurgical biopsies [28].

## 5. Conclusions

Our results demonstrated the feasibility of a simple, static [^18^F]F-DOPA PET/CT acquisition (lasting less than 5 min and performed 1.5 min after the injection) for establishing a differential diagnosis between RN and MP.

## Figures and Tables

**Table 1:** Demographic and baseline clinical characteristics of the study population.

Variable	Categories	Subjets Included, n (%) or Mean ± SD
Sex	Male	6 (40%)
	Female	9 (60%)
Age (years)		67.5 ± 11.3
Body mass index (kg/m^2^)		26.6 ± 4.1
Primary tumor	Lung	7 (47%)
	Breast	3 (20%)
	Melanoma	2 (13%)
	Kidney	1 (7%)
	Undetermined	2 (13%)
Lesion site	Supratentorial	11 (73%)
	Infratentorial	4 (27%)
Time interval between MRI and PET (days)		45.3 ± 35.0
Total number of lesions		19

**Table 2:** Comparative analysis of the RN and MP groups with regard to the LS_max_/CL_mean_ [^18^F]F-DOPA ratio for the V120, V270, and Vclin volumes.

PET Volume	Patient Group	Median [IQR ^1^]	Mean ± SD ^2^	Difference [95% CI ^3^]
V120	RN	2.19 [1.61; 2.59]	2.22 ± 0.82	−2.91 ^5^ [−7.17; −1.40]
	MP	4.87 [3.84; 8.35]	6.22 ± 3.13	
V270	RN	1.96 [1.55; 2.22]	2.01 ± 0.70	−2.77 ^5^ [−6.22; −1.33]
	MP	4.56 [3.59; 7.43]	5.85 ± 3.12	
Vclin	RN	1.95 [1.46; 2.12]	1.89 ± 0.53	−1.13 ^4^ [−2.81; −0.43]
	MP	2.7 [2.65; 3.95]	3.41 ± 1.32	

Note: ^1^ interquartile range, ^2^ standard deviation, ^3^ confidence interval, ^4^ *p* < 0.05, ^5^ *p* < 10^−3^.

**Table 3:** Diagnostic performance of the *LS_max_*/*CL_mean_* ratio for the V120, V270, and Vclin volumes.

PET Volume	Accuracy (%)	Cutoff	Sensitivity (%)	Specificity (%)	AUC ^1^ (%)
V120	94.7	3.16	100	91.7	97.6
V270	94.7	3.33	85.7	100	97.6
Vclin	84.2	2.22	100	75	90.5

Note: ^1^ area under the curve.

## Data Availability

The datasets used and/or analyzed during the current study are available from the corresponding author upon reasonable request.

## Abbreviations

[^18^F]F-DOPA	3,4-dihydroxy-6-[^18^F]fluoro-L-phenylalanine
AUC	Area under the curve
BM	Brain metastasis
CE-MRI	Contrast-enhanced magnetic resonance imaging
*COV*	Coefficient of variation
LM	List mode
LS	Lesion
LYSO	Lutetium–yttrium–orthosilicate
MP	Metastasis progression
RN	Radiation necrosis
SiPM-PET	Silicon photomultiplier-based PET scanner
SD	Standard deviation
